# The Effect of Deep Brain Stimulation on Sleep and Cognition in Patients With Parkinson's Disease

**DOI:** 10.1111/jsr.70193

**Published:** 2025-08-28

**Authors:** Monique E. H. Valk‐Geuke, Gert J. Geurtsen, Rob M. A. de Bie, Rick Schuurman, Martijn Beudel, Esmée Verwijk

**Affiliations:** ^1^ Department of Medical Psychology Amsterdam UMC Amsterdam the Netherlands; ^2^ Department of Medical Psychology Amsterdam UMC, Amsterdam Neuroscience Amsterdam the Netherlands; ^3^ Department of Neurology Amsterdam UMC, Amsterdam Neuroscience Amsterdam the Netherlands; ^4^ Department of Neurosurgery Amsterdam UMC Amsterdam the Netherlands; ^5^ Department of Psychology, Brain and Cognition University of Amsterdam Amsterdam the Netherlands

**Keywords:** cognition, deep brain stimulation, Parkinson, sleep, sleepiness

## Abstract

Sleep–wake disturbances and cognitive decline are among the most common nonmotor symptoms of Parkinson's disease (PD). Deep brain stimulation (DBS) can successfully alleviate motor symptoms. However, the impact on sleep–wake disturbances and cognitive decline, and their interaction, is yet unclear. We aim to investigate changes in and interaction between subjective sleep and cognition following DBS. We performed a study on data from the Amsterdam‐PD‐DBS database with assessments at baseline and at 6 months post‐operative. Subjective sleep was assessed with the sleep and sleepiness items of the Movement Disorder Society‐Unified Parkinson's Disease Rating Scale. Cognition was assessed with neuropsychological tests for the domains of language, processing speed, executive functioning, and memory. Three hundred and sixty‐five PD patients were included. Subjective sleep and sleepiness significantly improved after DBS. The proportion of patients with clinically relevant sleep disturbances dropped significantly from 76.4% to 50.1% (*p* < 0.001). Significant declines were observed in verbal fluency (*p* < 0.001, *p =* 0.005), processing speed (*p* < 0.001), and executive function (*p* < 0.001, *p =* 0.013), while delayed memory showed a significant improvement (*p =* 0.025). Significant associations were found for reduction in sleepiness and less decline in category fluency (*p* = 0.014) while no significant relationship between changes in sleep and changes in cognitive outcomes was present. This study provides strong evidence for the beneficial effects of DBS on sleep and sleepiness. No evidence was found for an association between reduction in subjective sleep disturbances following DBS and change in cognition.

## 1 Introduction

Parkinson's disease (PD) is one of the most rapidly growing neurological disorders and in 2016 the number of people with PD reached 6.1 million globally (GBD 2015 Neurological Disorders Collaborator Group 2017). The motor symptoms of PD are characterised by tremor, rigidity, bradykinesia/akinesia and postural instability. However, nonmotor symptoms such as sleep–wake disturbances, cognitive impairment, sensory symptoms, cardiovascular symptoms and neuropsychiatric symptoms (e.g., depression, anxiety, apathy and hallucinations) are also cardinal symptoms of the disease (Balestrino and Schapira 2020).

Sleep–wake disturbances are among the most common nonmotor symptoms of PD. The lifetime prevalence of sleep–wake disturbances in individuals with PD varies between 74% and 98% (Bailey et al. 2021). Significant reductions in subjective sleep quality as well as objective sleep quality such as decreased total sleep time and increased wake time after sleep onset are present in PD (Asadpoordezaki et al. 2025; Zhang et al. 2020). These sleep–wake disturbances have a significant impact on quality of life, motor symptom severity and other nonmotor symptoms (Asadpoordezaki et al. 2025; Chahine et al. 2017). Cognitive decline is another common nonmotor symptom, leading to global, neuropsychiatric, psychosocial and quality‐of‐life deterioration (Simon‐Gozalbo et al. 2020). Cognitive decline in PD is more common in the domains of executive function, attention, visuospatial abilities and memory (Aarsland et al. 2017). However, cognitive profiles in PD are heterogeneous, ranging from patients with normal performance to patients with impaired performance on different cognitive domains (Dujardin et al. 2013). Sleep–wake disturbances and cognitive impairment in PD patients appear to be related. Sleep disorders in PD patients, such as REM sleep behaviour disorder (RBD), obstructive sleep apnea (OSA), restless legs syndrome (RLS) and excessive daytime sleepiness (EDS), are associated with cognitive deficits in memory, executive functioning, attention, language and visuospatial abilities (Maggi et al. 2021). Furthermore, sleep disorders in PD patients seem to increase the risk for future cognitive decline (Maggi et al. 2021). However, these sleep disorders differ in their risk profiles for cognitive decline. While RBD is a well‐established risk factor (Maggi et al. 2021), a causal relationship between RLS and cognitive impairment has not been established (Cederberg et al. 2020).

The pathological hallmark of PD is the loss of dopaminergic neurons with subsequent depigmentation of the pars compacta of the substantia nigra and the presence of Lewy bodies (Balestrino and Schapira 2020). However, throughout the disease course, Lewy body pathology becomes more widespread in the nervous system with a special correlation with the severity of nonmotor symptoms. Current medical treatment is based on replacement of dopamine, with levodopa as the gold standard for the treatment of PD (Balestrino and Schapira 2020). When pharmacotherapy becomes insufficient, advanced therapies such as deep brain stimulation (DBS) may be considered. DBS is a neurosurgical procedure involving the implantation of electrodes in specific brain regions to modulate neural activity (Malvea et al. 2022). DBS can successfully alleviate the motor symptoms of PD and reduce the amount of medication. However, the impact on nonmotor symptoms, such as sleep–wake disturbances and cognitive decline, is yet unclear, with various studies showing conflicting results (Malvea et al. 2022).

Most studies on the efficacy of DBS on sleep show some objective improvement in sleep in PD patients following DBS, such as increased total sleep time, decreased total wake time after sleep onset, longer periods of continuous sleep (Bailey et al. 2021) and subjective sleep quality improvement (Jung et al. 2021; Kurtis et al. 2017). Furthermore, controlled prospective studies show subjective sleep quality improvement following DBS (Kurtis et al. 2017). The impact of DBS on RLS is also positive while no effect is seen on RBD (Bailey et al. 2021). However, a recent systematic review and meta‐analysis on 26 studies performed in PD patients with STN‐DBS reported mixed findings on the efficacy of DBS on sleep (Wadhwa et al. 2024). Furthermore, DBS studies on daytime sleepiness are inconclusive (Jung et al. 2021; Kurtis et al. 2017; Zuzuarregui and Ostrem 2020).

Studies on the impact of DBS on cognitive performance show inconsistent outcomes. So far, the strongest evidence is present for a slight decline in language function, specific in verbal fluency (Racki et al. 2022; You et al. 2020). However, inconclusive evidence is present for decline in executive function, memory, attention and processing speed (Racki et al. 2022). Global cognition does not appear to be significantly impaired by DBS (Racki et al. 2022). Although sleep disturbances are associated with cognitive deficits in PD patients, none of the studies investigated the potential association between sleep–wake disturbances and cognitive decline following DBS. While dopaminergic treatments, like levodopa, can have negative effects on both cognition and sleep (Balestrino and Schapira 2020; Chang et al. 2019; Roy et al. 2018; Schaeffer et al. 2021), and typically decrease following DBS, the levodopa equivalent daily dose (LEDD) is an important factor to consider.

Analysing the association between sleep and cognition before and following DBS can serve as an initial step towards comprehending the complex relationship between sleep, cognition and DBS in PD patients. Understanding how sleep, cognition and DBS interact can serve as a foundation for developing more personalised‐based treatment options in which clinicians tailor interventions based on the specific needs and characteristics of each patient.

With the current study we aim to investigate whether there is an association between sleep and/or sleepiness and cognition following DBS. Based on the literature we expect that PD patients show improvement in subjective sleep and a decline in cognition following DBS (Bailey et al. 2021; Kurtis et al. 2017; Racki et al. 2022). Expectations regarding the effect of DBS on sleepiness cannot be drawn so far. Since sleep–wake disturbances, including EDS, have a negative impact on cognition we expect that improvement in subjective sleep and/or sleepiness following DBS is associated with less cognitive decline.

## 2 Methods

### 2.1 Study Design and Participants

We used data from the Amsterdam PD DBS database, a monocentric database that constitutes all PD patients treated with DBS at Amsterdam UMC since the year 2000 (Swinnen et al. 2024). In addition to demographics, the database contains multimodal patient information at different DBS stages. Database search was performed on 10 November 2023. Data was only extracted from the database for the period from 2014 to 2023, as systematic follow‐up data of the entire movement disorder society‐unified Parkinson's disease rating scale (MDS‐UPDRS), one of the outcomes for this study, was only available from 2014 onwards.

Patients were included in this study if they were aged 18 years or older, had idiopathic PD, and underwent bilateral STN DBS. Patients were excluded if they did not agree to their data being used for secondary research, had undergone previous stereotactic surgery and/or were diagnosed with (Parkinson) dementia. The surgical procedure had been described in detail elsewhere (Holewijn et al. 2021).

Patients were only included for analysis when MDS‐UPDRS measures for sleep and sleepiness (item 1.7 and 1.8) before (= baseline) and at 6 months post‐operative evaluation were available. For these patients, demographics and clinical data were retrieved at baseline, which took place approximately 2 months prior to surgery, including information on age, sex, education (Verhage 1964), PD duration, Hoehn and Yahr scores (Hoehn and Yahr 1967), MDS‐UPDRS scores (Goetz et al. 2008), LEDD, the use of sleep‐related medication and neuropsychological test scores. At 6 months post‐operative evaluation, MDS‐UPDRS measures for sleep and sleepiness and neuropsychological test scores as well as LEDD were retrieved.

### 2.2 Study Outcomes

#### 2.2.1 Subjective Sleep and Sleepiness

Items 1.7 ‘sleep problems’ and 1.8 ‘daytime sleepiness’ of the MDS‐UPDRS were used to assess the degree of sleep disturbances on a scale from 0 (no problems) to 4 (severe problems) at baseline and at 6 months post‐operative evaluation. These items are effective in screening for sleep disturbances and daytime sleepiness in PD patients (Horvath et al. 2014). Based on the MDS‐UPDRS 1.7 score, patients were divided into two groups, namely those with and those without clinically relevant sleep disturbances at baseline. Clinically relevant sleep disturbances were defined as a MDS‐UPDRS sub‐item 1.7 score ≥ 2. A score of 2 corresponds to ‘mild’ sleep problems, which usually cause some difficulties getting a full night of sleep and is considered therefore clinically relevant in recent studies (Peball et al. 2022; Rutten et al. 2017; Xu et al. 2021).

#### 2.2.2 Cognition

Neuropsychological assessments were conducted at baseline for all patients. Six months post‐operative evaluation of cognitive data were available only for a subset of patients, namely those enrolled in the GALAXY trial (Holewijn et al. 2021) and those (a small subset of patients) who underwent follow‐up testing based on clinical indication, for example after DBS reprogramming, cognitive complaints or apathy. As such, cognitive data on 6 months post‐operative evaluation were not available for the full cohort. Phonemic fluency was assessed with the controlled oral word association test (COWAT) (Rosen 1980), and semantic fluency with the category fluency (Rosen 1980). Attention and processing speed was tested with the Stroop colour‐word test (Stroop)‐I, Stroop‐II (Stroop 1935)) and the trail making test (TMT)‐A (Reitan 1992). Executive functioning was measured by the Stroop III and TMT‐B. The Dutch version of Rey's auditory verbal learning test (RAVLT) was used to assess immediate and delayed recall (Rey 1958). Higher raw scores on COWAT, category fluency, and RAVLT indicate better performance, while higher raw scores on the Stroop I, II, III and TMT A and B indicate worse performance.

### 2.3 Statistical Analysis

A descriptive analysis of demographic and clinical data at baseline was performed in the included patients for the full data set, as well as for the group with clinically relevant and without clinically relevant sleep disturbances. Normality of distribution of clinical scores was tested with the Shapiro–Wilk method. For baseline difference between the group with clinically relevant sleep disturbances and the group without clinically relevant sleep disturbances, independent *t*‐test, Mann–Whitney U test, and Chi‐square test were used appropriately. To examine the change in sleep, sleepiness and cognition before STN DBS and 6 months post‐operatively for the complete group, we used paired‐samples *t*‐test for normally distributed variables and the Wilcoxon Signed‐Rank test for ordinal variables and for non‐normally distributed variables. A Chi‐square test was used to analyse whether the proportion of PD patients with clinically relevant sleep disturbances differed between baseline and 6 months post‐operative evaluation.

In the subgroup of patients who underwent neuropsychological testing at baseline and at 6 months post‐operative evaluation, simple linear regression was used to assess the relation between subjective sleep/sleepiness and cognitive decline following DBS, with the change in subjective sleep or change in subjective sleepiness as the predictor and the change in cognitive outcome measures as the dependent variables. Only cognitive measures that showed a significant change following DBS were selected as outcome measures, as these identify the most sensitive cognitive measures for change following DBS in this database.

Dopaminergic medication was converted to LEDD (Tomlinson et al. 2010) and prior to the analysis, an independent *t*‐test was conducted to compare PD patients with clinically relevant sleep disturbances to PD patients without clinically relevant sleep disturbances in terms of changes in LEDD from baseline to 6 months post‐operative evaluation. If these groups differ significantly on changes in LEDD, then LEDD was included in the linear regression model as a covariate factor.

All statistical analyses were performed in IBM SPSS Statistics for Windows, version 28.0.1.0 (IBM Corp, Armonk, NY). Statistical significance was set at a two‐sided 5% *α*‐level.

## 3 Results

The database consisted of 710 patients who underwent DBS during the period 2014–2023. Patients who did not have a diagnosis of PD (*n* = 2), were diagnosed with a (Parkinson) dementia (*n* = 4) were implanted in a target other than the STN (*n* = 4), underwent unilateral STN implantation (*n* = 8), only changed their DBS battery (*n* = 1) and/or did not agree to their data being used for secondary research (*n* = 17) were removed from the database, leaving a total of 676 patients in the database (total number of excluded patients is lower than the sum due to overlapping criteria). Of those patients, 311 were excluded because of missing values on items 1.7 and/or 1.8.

Included patients differed significantly at baseline from excluded patients on age, Hoehn & Yahr score OFF and ON, MDS‐UPDRS 3 ON, LEDD, subjective sleep disturbance, Category fluency, COWAT, Stroop I, II and III and TMT A and B (See Table S1).

Of the remaining 365 PD patients, a total of 279 (76.4%) patients were, based on the MDS‐UPDRS 1.7 score, categorised as having clinically relevant sleep disturbances. Except for a higher LEDD, a shorter disease duration, more subjective sleep problems and more subjective sleepiness (resp. *t*(357) = 1.97, *p* = 0.050, *d* = 0.25; *U* = 10,231, *z* = −2.03, *p* = 0.043, *r* = −0.11; *U* = 0, *z* = −14.63, *p* < 0.001, *r* = −0.77; and *U* = 7815, *z* = −4.16, *p* < 0.001, *r* = −0.22), the patients with clinically relevant sleep disturbances were comparable to patients without clinically relevant sleep disturbances in terms of baseline demographic and clinical characteristics. More details of the demographic and clinical characteristics are shown in Table 1.

**TABLE 1:** Characteristics of the study population at baseline.

		Full data set (*n* = 365)		PD patients with clinically relevant sleep disturbances (MDS‐UPDR 1.7 ≥ 2, *n* = 279)		PD patients without clinically relevant sleep disturbances (MDS‐UPDR 1.7 < 2, *n* = 86)
	*n*		*n*		*n*	
Age (in years)	365	65.00 (12.00)	279	65.00 (13.00)	86	65.50 (10.00)
Females	365	130 (35.6%)	279	97 (34.8%)	86	33 (38.4%)
Education (Verhage^a^)	215	5 (1.00)	160	5.00 (1.00)	55	6.00 (1.00)
Disease duration (in years)	364	9.00 (5.00)	278	9.00 (6.00)*	86	10.00 (6.00)*
H&Y OFF (1–5 severe)	138	2.00 (1.00)	108	2.00 (1.00)	30	2.00 (0.25)
H&Y ON (1–5 severe)	57	2.00 (0.00)	46	2.00 (0.00)	11	2.00 (1.00)
MDS‐UPDRS total score 3 OFF	360	47 (19.00)	274	46.00 (19.25)	86	48 (18.00)
MDS‐UPDRS total score 3 ON	348	18 (15.00)	270	18.00 (14.25)	84	18 (14.75)
LEDD	359	1407.47 ± 655.12	275	1444.87 ± 664.36*	84	1285.04 ± 611.82*
Use of sleep related medication	365	82 (22.5%)	279	69 (24.7%)	86	13 (15.1%)
Subjective sleep						
MDS‐UPDRS 1.7 (sleep)	365	3.00 (1.00)	279	3.00 (1.00)*	86	1.00 (1.00)*
MDS‐UPDRS 1.8 (sleepiness)	365	2.00 (1.00)	279	2.00 (1.00)*	86	1.00 (2.00)*
Language: fluency						
Category fluency	229	41.00 (15.50)	171	41.00 (15.00)	58	41.00 (15.25)
COWAT	229	38.44 ± 12.91	171	38.92 ± 12.94	58	37.03 ± 12.85
Attention and processing speed						
Stroop I	232	44.00 (12.00)	173	44.00 (14.00)	59	44.00 (12.00)
Stroop II	231	60.00 (16.00)	172	60.00 (18.00)	59	62.00 (12.00)
TMT A	232	33.50 (18.00)	173	32.00 (19.00)	59	36.00 (16.00)
Executive function						
Stroop III	231	102.00 (36.00)	172	101.00 (41.50)	59	106.00 (30.00)
TMT B	231	88.00 (61.00)	172	87.50 (59.75)	59	93.00 (79.00)
Memory						
RAVLT immediate recall	230	42.30 ± 10.81	172	42.45 ± 11.10	58	41.83 ± 9.99
RAVLT delayed recall	229	9.00 (5.00)	171	9.00 (5.00)	58	9.00 (4.00)

*Note*: Data are presented as mean ± standard deviation for normally divided continuous variables, median (IQR) for non‐normally divided continuous variables and ordinal variables, and number (percent) for categorical variables.

Abbreviations: COWAT, controlled oral word association test; H&Y, Hoehn & Yahr stage; LEDD, Levodopa equivalent daily dose; MDS‐UPDRS 1.7, degree of sleep disturbances; MDS‐UPDRS 1.8, daytime sleepiness; MDS‐UPDRS, movement disorder society—unified Parkinson's disease rating scale; RAVLT, Rey's auditory verbal learning test; Stroop, Stroop colour‐word test; TMT, trail making test.

* Indicates a significant difference (*p* < 0.05) between ‘PD patients with clinical relevant sleep problems’ and ‘PD patients without clinical relevant sleep problems’ at baseline.

^a^ Verhage: Level of education which is commonly used in the Netherlands (Verhage 1964), coded on a seven‐point scale on which one stands for ‘no education’ and seven stands for ‘university degree’.

LEDD decreased 57.7% from 1407.47 ± 655.12 mg to 595.95 ± 362.25 mg 6 months post‐operatively. As this reduction does not significantly differ between the PD patients with clinically relevant sleep problems and the PD patients without clinically relevant sleep problems (*t*(345) = −0.558, *p* = 0.577) LEDD was not included in regression analysis.

### 3.1 Six Months Post‐Operative Change in Sleep, Sleepiness and Cognition

PD patients reported a 6 months post‐operatively significant improvement in subjective sleep and sleepiness (Table 2). Furthermore, 6 months post‐operatively the percentage of patients with clinically relevant sleep problems was reduced from 76.4% to 50.1%, a significant reduction, *χ* ^2^ (1, *N* = 365) = 37.76, *p* < 0.001.

**TABLE 2:** Six months post operatively change in sleep, sleepiness and cognition.

	Baseline		Six months post‐operative				
	*n*	M ± SD/Mdn (IQR)	*n*	M ± SD/Mdn (IQR)	*t*/*Z*	*p*	*d*/*r*
Subjective sleep							
MDS UPDRS 1.7 (sleep)	365	3.00 (1.00)	365	1.00 (3.00)	−9.90	**< 0.001**	−0.62
MDS UPDRS 1.8 (sleepiness)	365	2.00 (1.00)	365	2.00 (2.00)	−4.12	**< 0.001**	−0.30
Language: fluency							
Category fluency	229	41.00 (15.50)	38	37.00 (9.00)	−4.19	**< 0.001**	−0.70
COWAT	98	38.50 ± 11.81	98	35.87 ± 12.43	2.84	**0.005**	0.29
Attention and processing speed							
Stroop I	232	44.00 (12.00)	99	48.00 (14.00)	4.76	**< 0.001**	−0.52
Stroop II	231	60.00 (16.00)	99	68.00 (18.00)	6.47	**< 0.001**	−0.67
TMT A	232	33.50 (18.00)	99	35.00 (23.00)	0.05	0.961	0.00
Executive function							
Stroop III	231	102.00 (36.00)	98	110.00 (49.00)	5.08	**< 0.001**	−0.52
TMT B	231	88.00 (61.00)	98	88.50 (68.50)	2.48	**0.013**	−0.25
Memory							
RAVLT immediate recall	98	41.27 ± 10.86	98	42.72 ± 11.79	−1.67	0.099	−0.17
RAVLT delayed recall	229	9.00 (5.00)	99	9.00 (5.00)	2.24	**0.025**	−0.25

*Note*: Data are presented as mean ± standard deviation, *t* and *d* for normally divided continuous variables and median (IQR), *Z* and *r* for non‐normally divided continuous variables and ordinal variables. Bold values indicate statistically significance (*p* < 0.05).

Abbreviations: COWAT, controlled oral word association test; IQR, interquartile range; M, mean; Mdn, median; MDS‐UPDRS 1.7, degree of sleep disturbances; MDS‐UPDRS 1.8, daytime sleepiness; MDS‐UPDRS, movement disorder society—unified Parkinson's disease rating scale; RAVLT, Rey's auditory verbal learning test; SD, standard deviation; Stroop, Stroop colour‐word test; TMT, trail making test.

For cognitive measures, PD patients showed a 6 months post‐operatively significant decline on category fluency, COWAT, Stroop I, Stroop II, Stroop III and TMT B. PD patients showed a 6 months post‐operatively significant improvement on the RAVLT delayed recall (Table 2).

### 3.2 The Influence of Change in Sleep on Cognition

Category fluency, COWAT, Stroop I, II and III, TMT B and RAVLT delayed recall showed a statistically significant 6 months post‐operatively change in our population and were therefore selected for linear regression analysis.

There was no significant relationship between changes in sleep and changes in cognitive outcomes (Table 3). The R squared value was also low for all cognitive outcomes, indicating that the model did not explain any amount of variability in changes in cognitive outcomes.

**TABLE 3:** Associations between changes in subjective sleep/sleepiness and cognitive outcomes.

Pre‐post operative cognitive change	Pre‐post operative sleep change			Pre‐post operative sleepiness change		
	*R*	*R* ^2^	*p*	*R*	*R* ^2^	*p*
Category fluency	−0.200	0.040	0.229	−0.395	0.156	**0.014**
COWAT	−0.124	0.015	0.225	0.024	0.001	0.813
Stroop I	0.118	0.014	0.246	0.101	0.010	0.320
Stroop II	0.087	0.007	0.394	0.162	0.026	0.109
Stroop III	0.024	0.001	0.818	0.156	0.024	0.126
TMT B	0.146	0.021	0.150	0.088	0.008	0.389
RAVLT delayed recall	−0.013	0.000	0.897	−0.084	0.007	0.413

*Note*: Bold values indicate statistically significance (*p* < 0.05).

Abbreviations: COWAT, controlled oral word association test; RAVLT, Rey's auditory verbal learning test; Stroop, Stroop colour‐word test; TMT, trail making test.

A significant negative association was found for change in subjective sleepiness and change in category fluency (*F*(1) = 6.64, *p* = 0.014). The *R* ^2^ value was 0.156, indicating that the model did explain a weak amount of the variability in changes in category fluency.

## 4 Discussion

The aim of the current study was to investigate changes in and interaction between sleep and/or sleepiness and cognition before DBS and 6 months post‐operatively. Firstly, consistent with previous research (Kurtis et al. 2017), our findings showed a significant improvement in subjective sleep and sleepiness following DBS. Not only did our patients report fewer sleep disturbances following DBS, but the proportion of patients in our database that met the criteria for clinically relevant sleep disturbance also dropped from 76.4% to 50.1%. Although this represents a significant reduction, it also indicates that after DBS still 50% of the patients experience sleep disturbances. Previous studies on subjective sleepiness following DBS were inconsistent. Our findings are in line with one previous study showing improvement in subjective sleepiness (Bargiotas et al. 2017). Other studies (Hjort et al. 2004; Lyons and Pahwa 2006) found no change in sleepiness, which cannot be explained by differences in sample size, study design or outcome measures. This issue merits further investigation.

Secondly, our findings showed significant changes in cognitive outcomes following DBS, which is in line with previous research (Racki et al. 2022; You et al. 2020). We found a significant decline in verbal fluency and in executive function. We found mixed results for processing speed, where two out of three outcome variables showed a decline. We found an improvement in delayed recall while no change was observed for immediate recall. This is not in line with previous studies in which no change or a slight decline in memory following DBS was reported (Racki et al. 2022). However, studies in which the decline was found had a longer follow‐up period, namely 1 to 3 years (Bucur and Papagno 2023), where the slight decline may be the effect of the natural course.

Finally, we found that a reduction in subjective sleepiness following DBS was associated with less cognitive decline in verbal fluency following DBS. Although the associations were small, they were significant. Contrary to our expectations, we did not find any association between the reduction in subjective sleep disturbances following DBS and the change in cognition following DBS.

The strength of this study is that our final cohort size is one of the largest in studies of this kind. Results of the present study must however be interpreted in the light of a number of limitations. We had to exclude nearly 50% of the patients from the original database, primarily due to logistical reasons, unrelated to clinical status. However, excluded patients differed from the included ones in terms of age, disease severity, medication use, and cognitive function, which limits generalizability and may introduce selection bias. Among the available cognitive data 6 months post‐operatively, a small number of patients underwent neuropsychological testing based on clinical indication, which may introduce some selection bias. However, given the limited size of this subset, we consider this impact on the overall findings to be minimal. Another limitation is the use of the MDS‐UPDRS to assess subjective sleep and sleepiness. Although proven to be effective for screening sleep disturbances and daytime sleepiness in PD patients (Horvath et al. 2014), its suitability as a valid instrument for assessing changes in sleep and sleepiness is uncertain. Other instruments like the Insomnia Severity Index (ISI) (Morin et al. 2011), the Pittsburgh Sleep Quality Index (PSQI) (Buysse et al. 1989) and the Parkinson Disease Sleeping Scale revised version (PDSS‐2) (Trenkwalder et al. 2011) for subjective sleep, and the Epworth Sleepiness Scale (ESS) (Johns 1991) for subjective sleepiness, which have demonstrated sensitivity for change, are more appropriate and should be incorporated in future research. Additionally, while we did not include a control group, our observations do not allow conclusions regarding causality of improving sleep following DBS or cognitive decline following DBS. Lastly, other factors, including progression of the disease itself, motor disturbances, the occurrence of sleep disorders such as obstructive sleep apnea, restless leg syndrome, rapid eye movement sleep behaviour disorder, the use of sleep medication, the occurrence of neuropsychiatric symptoms and the use of concomitant medication have not been controlled for. Future prospective studies should incorporate such additional factors to more comprehensively evaluate determinants of cognitive change following DBS.

The current study focused on subjective sleep, which is not necessarily correlated with objective sleep disturbances (Cudney et al. 2022). Future research at the association between sleep and cognition following DBS should also incorporate objective sleep measures. Electrophysiologic recordings from DBS electrodes, in the form of local field potentials (LFPs) may offer a promising method to incorporate objective sleep measures in future studies. Multiple studies have already shown the feasibility of using LFPs to determine sleep stages for extended periods during both the inactive and active states of DBS (Baumgartner et al. 2021). Furthermore, it is also important to differentiate between sleep disturbances and sleep disorders. While our study did not find an association between changes in sleep disturbances and cognitive decline following DBS, previous research showed an association between sleep disorders, namely RBD, OSA, RLS and EDS, and cognitive decline (Maggi et al. 2021). Future research should focus on the effect of active treatment of various sleep disorders in PD patients and the impact on cognitive decline, in which not only the severity of sleep problems is taken into account but also the underlying sleep disorder.

## 5 Conclusion

In conclusion, our study provides strong evidence for improvement of subjective sleep disturbances and sleepiness following DBS. Despite this improvement, the prevalence of clinically relevant sleep disturbances following DBS is still high, and this highlights the importance of assessment and treatment of sleep disturbances and sleepiness in PD patients to enhance the overall quality of life for these patients. We did not find an association between the reduction in subjective sleep disturbances following DBS and change in cognition following DBS. Further research in understanding the complex relationship between DBS, sleep and cognition in PD patients is necessary.

## Author Contributions

**Monique E. H. Valk‐Geuke:** conceptualization, methodology, formal analysis, writing – original draft, investigation. **Gert J. Geurtsen:** conceptualization, writing – review and editing. **Rob M. A. de Bie:** writing – review and editing. **Rick Schuurman:** writing – review and editing, project administration. **Martijn Beudel:** writing – review and editing. **Esmée Verwijk:** supervision, writing – review and editing, conceptualization, methodology.

## Disclosure

The authors have nothing to report.

## Ethics Statement

In this retrospective study, data was collected from a clinical database from patients who received DBS in the Amsterdam University Medical Centre between 2014 and 2023 in a care as usual setting and were informed about the secondary use of their clinical data. In this passive consent procedure, patients had the possibility to opt out, to exclude their clinical data for scientific purposes. This retrospective study was assessed by the local ethical board and not classified as medical research involving human subjects.

## Conflicts of Interest

The authors declare no conflicts of interest.

## Supporting information


**Table S1:** Characteristics of the study population at baseline: included versus excluded patients.

## Data Availability

The data that support the findings of this study are available from MB upon reasonable request.
