# Supplementary material for: The Effect of Deep Brain Stimulation on Sleep and Cognition in Patients With Parkinson's Disease
Source: J Sleep Res. 2025 Aug 28;35(2):e70193. doi: 10.1111/jsr.70193 (PMC13003266; doi:10.1111/jsr.70193)
Supplement: Supplementary file 1 — Table S1: Characteristics of the study population at baseline: included versus excluded patients. [file JSR-35-e70193-s001.docx]

**Supplementary material**

**Table S1**
*Characteristics of the study population at baseline: included versus excluded patients*

|  |  | Included patients (n = 365) |  | Excluded patients (n = 345) |  |  |
| --- | --- | --- | --- | --- | --- | --- |
|  | *n* |  | *n* |  | *t* / *Z /* χ^2^ | *P* |
| Age (in years) | 365 | 65.00 (12.00) | 340 | 68.00 (13.00) | -3.82 | **<0.001** |
| Females | 365 | 130 (35.6%) | 344 | 131 (38.1%) | 0.50 | 0.533 |
| Education (Verhage^a^) | 215 | 5.00 (1.00) | 48 | 5.00 (2.00) | -1.37 | 0.172 |
|  |  |  |  |  |  |  |
| Disease duration (in years) | 364 | 9.00 (5.00) | 313 | 9.00 (6.00) | -0.14 | 0.893 |
| H&Y OFF (1-5 severe) | 138 | 2.00 (1.00) | 139 | 3.00 (1.00) | -2.98 | **0.003** |
| H&Y ON (1-5 severe) | 57 | 2.00 (0.00) | 76 | 2.00 (1.00) | -2.50 | **0.014** |
| MDS-UPDRS total score 3 OFF | 360 | 47.00 (19.00) | 263 | 49.00 (22.00) | -0.78 | 0.436 |
| MDS-UPDRS total score 3 ON | 354 | 18.00 (15.00) | 261 | 23.00 (17.00) | -4.32 | **<0.001** |
| LEDD | 359 | 1350.00 (795.00) | 280 | 1263.88 (876.19) | -2.87 | **0.004** |
| Use of sleep related medication | 365 | 82 (22.5%) | 345 | 73 (21.2%) | 0.18 | 0.716 |
|  |  |  |  |  |  |  |
| *Subjective sleep* |  |  |  |  |  |  |
| MDS-UPDRS 1.7 (sleep) | 365 | 3.00 (1.00) | 208 | 2.00 (2.00) | -2.84 | **0.005** |
| MDS-UPDRS 1.8 (sleepiness) | 365 | 2.00 (1.00) | 211 | 2.00 (1.00) | -0.30 | 0.761 |
|  |  |  |  |  |  |  |
| *Language: fluency* |  |  |  |  |  |  |
| Category fluency | 229 | 41.00 (15.50) | 61 | 39.00 (13.50) | -2.24 | **0.025** |
| COWAT | 229 | 38.44 + 12.91 | 61 | 33.28 + 13.76 | 2.74 | **0.007** |
|  |  |  |  |  |  |  |
| *Attention and processing speed* |  |  |  |  |  |  |
| Stroop I | 232 | 44.00 (12.00) | 65 | 46.00 (16.00) | -2.64 | **0.008** |
| Stroop II | 231 | 60.00 (16.00) | 65 | 64.00 (18.69) | -2.36 | **0.018** |
| TMT A | 232 | 33.50 (18.00) | 65 | 43.00 (29.00) | -3.52 | **<0.001** |
|  |  |  |  |  |  |  |
| *Executive function* |  |  |  |  |  |  |
| Stroop III | 231 | 102.00 (36.00) | 65 | 112.00 (60.00) | -2.24 | **0.025** |
| TMT B | 231 | 88.00 (61.00) | 64 | 123.00 (150.75) | -3.44 | **<0.001** |
|  |  |  |  |  |  |  |
| *Memory* |  |  |  |  |  |  |
| RAVLT immediate recall | 230 | 42.30 + 10.81 | 64 | 39.50 + 10.88 | 1.83 | 0.069 |
| RAVLT delayed recall | 229 | 9.00 (5.00) | 64 | 8.00 (4.00) | -1.39 | 0.164 |
|  |  |  |  |  |  |  |

*^a^Verhage: Level of education which is commonly used in the Netherlands (Verhage, 1964), coded on a seven-point-scale on which one stands for “no education” and seven stands for “university degree*

*Data are presented as mean + standard deviation and t for normally divided continuous variables, median (IQR) and Z for non-normally divided continuous variables and ordinal variables, and number (percent) and* χ^2^ *for categorical variables. Abbreviations: MDS-UPDRS, Movement Disorder Society - Unified Parkinson's Disease Rating Scale; H&Y, Hoehn & Yahr stage; LEDD, Levodopa equivalent daily dose; MDS-UPDRS 1.7: degree of sleep disturbances; MDS-UPDRS 1.8: Daytime sleepiness; COWAT, Controlled Oral word Association Test; Stroop, Stroop color-word test; TMT, Trail Making Test; RAVLT, Rey's Auditory Verbal Learning Test*
